# Resumé of Our Actual Knowledge of the Movements of the Pupil in Health and Disease

**Published:** 1885-10

**Authors:** 


**Riassunto delle Attuali Nostre Cognizioni sui Movimenti della Pupillu nello Stato Fisiologico e Morboso.** (Resumé of our Actual Knowledge of the Movements of the Pupil in Health and Disease.) By Prof. ANTONIO QUAGLINO, of Pavia.

